# Association between visceral adiposity index, lipid accumulation products, and frailty in older Americans: the 2007–2018 NHANES cross-sectional study

**DOI:** 10.3389/fmed.2025.1541761

**Published:** 2025-03-26

**Authors:** Jie Xu, Zijuan Cai, Min Sun, Meng Chen, Yong Hu, Xiaobing Luo

**Affiliations:** ^1^Department of Sports Medicine, Sichuan Provincial Orthopedics Hospital, Chengdu, China; ^2^Chengdu International Studies University, Chengdu, China; ^3^Department of Knee Sports Injury, Sichuan Provincial Orthopedics Hospital, Chengdu, China; ^4^Department of Emergency Medicine, Nanchong Hospital of Traditional Chinese Medicine, Nanchong, China

**Keywords:** visceral adiposity index, lipid accumulation products, frailty, obesity, NHANES, cross-sectional study

## Abstract

The aim of this study was to investigate the relationship between visceral adiposity index (VAI) and lipid accumulation products (LAP) and frailty index (FI) in older Americans. Based on data from the 2007–2018 National Health and Nutrition Examination Survey (NHANES), the study population consisted of 3,396 older adults aged 60 years and older with a mean age of 69.48 ± 6.76 years. The findings of weighted multivariate regression analysis demonstrated a strong correlation between the prevalence of frailty prevalence and greater VAI and LAP. In the fully adjusted model, the association of VAI with frailty remained significant, with an OR of 1.49 (95% CI: 1.26, 1.77; *p* < 0.0001.) The association of LAP with frailty was also significant, with an OR of 1.88 (95% CI: 1.55, 2.29; *p* < 0.0001). Further nonlinear analyses by generalized additive modeling (GAM) revealed significant nonlinear relationships between VAI and LAP and frailty, and the nonlinear effects were more pronounced in the female population. Subgroup analyses showed that the positive correlations between VAI and LAP and frailty were generalized across populations and there was no significant interaction in most subgroups. In addition, sensitivity analyses validated the robustness of these results, further confirming the conclusion of VAI and LAP as independent risk factors for frailty. Finally, ROC analysis showed that LAP outperformed VAI in predicting frailty, suggesting the potential of LAP in early screening for frailty. Overall, VAI and LAP are independent risk factors for frailty in the elderly population and have important clinical applications.

## 1 Introduction

Frailty is a multidimensional health state characterized by an overall decline in physical, psychological and social functioning (1). The occurrence of frailty is closely related to a number of factors, particularly the distribution of body fat. As the global aging problem intensifies, frailty has emerged as a significant public health issue that impacts the well-being of the aging population, particularly in advanced countries like the U.S., where the prevalence of frailty in the elderly continues to rise (2). Therefore, identifying risk factors associated with frailty and adopting effective interventions have become important goals for improving the health of older adults.

A significant risk factor for frailty is thought to be an excessive buildup of visceral fat (3). VAI and LAP are indicators proposed in recent years for assessing abdominal obesity and fat distribution and are widely used in the study of metabolic diseases. VAI integrates parameters such as body mass index (BMI), waist circumference (WC), triglycerides (TG), and high-density lipoproteins (HDL-C), and can more accurately reflect the level of visceral fat and its metabolic risk (4). LAP is calculated by the combination of waist circumference and triglycerides, which can better reflect the level of abdominal fat accumulation (5). VAI and LAP are important in cardiovascular disease (6, 7), diabetes mellitus (8, 9), atherosclerosis (10, 11), chronic kidney disease (12, 13), and other diseases that have been widely used in research. The interaction of inflammatory states and metabolic disorders is recognized as one of the main drivers of weakness. Abdominal fat accumulation, as a metabolically active tissue, is capable of secreting pro-inflammatory factors, such as TNF-α and IL-6, which trigger systemic chronic low-grade inflammation, which in turn accelerates the loss of muscle mass and promotes the development of frailty (14). In addition, abdominal fat accumulation is strongly associated with insulin resistance (15), further exacerbating metabolic disorders such as diabetes and hyperlipidemia and enhancing the risk of frailty (16, 17). These studies suggest that VAI and LAP are potential risk factors associated with the development of frailty, providing theoretical support for exploring the relationship between VAI and LAP and frailty.

Despite the significant clinical implications of VAI and LAP, studies on the association between VAI and LAP and frailty are still relatively scarce. Therefore, the present study, based on data from the NHANES from 2007 to 2018, utilized a cross-sectional study design with the aim of evaluating the relationship between VAI and LAP and frailty, and providing a scientific basis for early screening, risk assessment, and intervention of frailty.

## 2 Materials and methods

### 2.1 Database sources and sample selection

**Figure 1:**
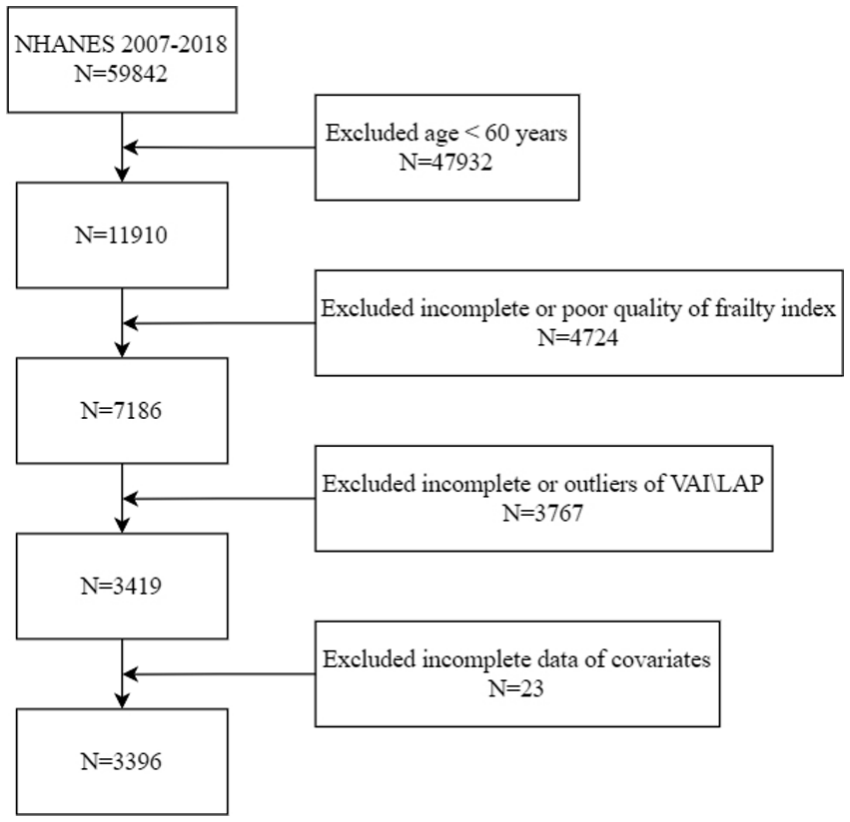
Flowchart of the population selection from NHANES.

The National Centre for Health Statistics (NCHS) administers the NHANES, a nationwide study that evaluates the nutrition and health of American adults living outside of institutions using a stratified multistage sampling method. The data from this survey are accessible to the public, and all participants have given their written consent. Selected data from NHANES between 2007 and 2018 were used for the analyses in this article. Initially, data from 59,842 participants from the NHANES 2007–2018 period were considered. The final sample included 3,396 participants, excluding 47,932 people under the age of 60, 4,724 persons with unreliable frailty index assessments, 3,767 persons with missing or outlier VAI and LAP data, and 23 people with missing covariates (Figure 1).

### 2.2 Assessment of frailty

Considering that the Frailty index (FI) index covers a more comprehensive range of health indicators, it is able to provide a comprehensive assessment of frailty in different aspects. A FI value greater than or equal to 0.25 is considered frailty (18). The Frailty Index consists of 49 factors, covering areas such as cognitive function, ability to perform daily activities, physical performance, chronic conditions, overall health, and laboratory test results. Supplementary Table 1 provides the full set of criteria. The severity of each criterion was assessed, with 0 indicating no frailty and 1 indicating severe frailty, and the frailty index was finally obtained by calculating the total score divided by the number of items (19, 20). To maintain the reliability of the data, only participants who completed 91% or more of the frailty assessment items were considered.

### 2.3 VAI and LAP assessment

VAI and LAP were used as exposure variables, and VAI was calculated as [WC/(36.58 + (1.89 × BMI))] × (TG/0.81) × (1.52/HDL) for women and [WC/[(39.68 + (1.88 × BMI))] × (TG/1.03) × (1.31/HDL)] for men (21). LAP was calculated as women (WC − 58) * TG, and male (WC − 65) * TG (22). BMI is in kg/m^2^, TG, HDL in mmol/L, and WC is in cm. All NHANES staff received rigorous training to ensure consistency and accuracy of measurements. To guarantee accuracy, the anthropometric apparatus at every Mobile Examination Centre was regularly calibrated and standardised. Logarithmic transformations of VAI and LAP were performed to correct for data skew and to standardize results. VAI and LAP data can be analyzed as either continuous or categorical variables (23). The Log VAI and Log LAP values were analyzed by dividing them into four groups (Log VAI first division: -1.84 < VAI ≤ -0.09; second division: -0.09 < VAI ≤ 0.40; third division: 0.40 < VAI ≤ 0.88; fourth division: 0.88 < VAI ≤ 2.45. Log LAP first division: 0.94 < LAP ≤ 3.39; second equal parts: 3.39 < LAP ≤ 3.86; third equal parts: 3.86 < LAP ≤ 4.33; fourth equal parts: 4.33 < LAP ≤ 5.84).

### 2.4 Covariates

To account for confounding factors, the study adjusted for several known covariates, including age, gender, race, education level, marital status, poverty income ratio (PIR), alcohol use, smoking, diastolic blood pressure (DBP), systolic blood pressure (SBP), energy intake, and Healthy Eating Index-2015 (HEI-2015). In addition, a history of chronic diseases such as diabetes, chronic obstructive pulmonary disease, cardiovascular illness, and chronic kidney disease, are also potential factors that affect frailty. Diabetes, COPD, and chronic kidney disease were identified through NHANES disease self-report data, i.e., by asking respondents if they had been told by a doctor that they had these diseases (24). Cardiovascular disease was determined by respondents’ self-report of having been diagnosed by a physician with heart disease, coronary heart disease, heart failure, or stroke. PIR were calculated as the ratio of household income to the U.S. federal poverty level, which is updated by the U.S. Department of Health and Human Services (25). Specific income levels are categorized as low income (PIR ≤ 1.3), moderate income (1.3 < PIR ≤ 3.5), and high income (PIR > 3.5), and several studies have used the same categorization (26, 27). Smoking was defined as consuming 100 or more cigarettes over one’s lifetime. Alcohol use is classified according to the current drinking status into five categories: never, former, heavy, moderate and mild drinking (28, 29). For detailed classification criteria, see Supplementary Document 2. At least three consecutive standard readings were averaged to estimate blood pressure. On the first day of the 24-h dietary recall study, dietary data were collected. The HEI-2015 evaluates a person’s dietary compliance with the Dietary Guidelines for Americans (30). Higher ratings indicate better food quality and healthier eating habits; the values range from 0 to 100.

### 2.5 Statistical analysis

Every statistical analysis applied the proper sampling weights and considered the intricate sampling design of NHANES. Categorical data are given as weighted proportions, whilst continuous variables are provided as mean ± standard error (SE). Weighted chi-square and t-tests were used to evaluate group differences at baseline. Model 1 (unadjusted), Model 2 (adjusted for age, gender, race, and education level), and Model 3 (further adjusted for variables such as marital status, PIR, smoking and alcohol consumption, SBP, DBP, HEI-2015, and energy intake) were the three weighted multivariate logistic regression models used to investigate the relationship between VAI and LAP and frailty. GAM were applied to investigate potential nonlinear relationships, while threshold effects and turning points were investigated using linear regression models. Subgroup analyses and interaction tests were performed as well. ROC analyses were used to compare the ability of VAI with LAP in frailty prediction. DeLong tests were conducted to assess statistical differences in the ROC analysis results. Sensitivity analyses consisted, among other things, of further adjusting for diabetes, chronic obstructive pulmonary disease, cardiovascular illness, and chronic kidney disease and estimating missing covariates using means. A two-tailed *p*-value was deemed statistically significant if it was less than 0.05. R software (version 4.4) and EmpowerStates (version 4.2) were used for all statistical studies.

## 3 Results

### 3.1 Baseline characteristics of participants

The study population’s basic characteristics are shown in Table 1. The study sample consisted of 3,396 individuals, with a mean age of 69.48 ± 6.76 years, 50.41% of whom were male and 49.59% of whom were female. 3.85 ± 0.70 was the mean LAP, and 0.40 ± 0.70 was the mean VAI. VAI and LAP were significantly higher in frailty patients compared to subjects without frailty (*p* < 0.001). Frailty patients were more likely to be female, older, less educated, widowed/divorced/separated, lower socio-economic status, higher BMI levels, non-alcohol drinkers, smokers, and to have diabetes, COPD and cardiovascular disease (all *p* < 0.05).

**Table 1:** The clinical characteristics of participants.

Characteristics	Total	Frailty		*p* value
	(*n* = 3,396)	NO (*n* = 2,328)	YES (*n* = 1,068)	
Age (years)	69.48 ± 6.76	68.89 ± 6.58	70.76 ± 6.98	<0.001
Gender %				<0.001
Female	1,684 (49.59%)	1,088 (46.74%)	596 (55.81%)	
Male	1712 (50.41%)	1,240 (53.26%)	472 (44.19%)	
Race %				0.099
Mexican American	381 (11.22%)	265 (11.38%)	116 (10.86%)	
Other Hispanic	361 (10.63%)	253 (10.87%)	108 (10.11%)	
Non-Hispanic White	1772 (52.18%)	1,199 (51.50%)	573 (53.65%)	
Non-Hispanic Black	629 (18.52%)	420 (18.04%)	209 (19.57%)	
Other Race	253 (7.45%)	191 (8.20%)	62 (5.81%)	
Education level %				<0.001
Less than 9th grade	424 (12.49%)	255 (10.95%)	169 (15.82%)	
9–11th grade	467 (13.75%)	283 (12.16%)	184 (17.23%)	
High school graduate	811 (23.88%)	537 (23.07%)	274 (25.66%)	
Some college or AA degree	925 (27.24%)	626 (26.89%)	299 (28.00%)	
College graduate or above	769 (22.64%)	627 (26.93%)	142 (13.30%)	
Marry %				<0.001
Married/Living with Partner	2,107 (62.04%)	1,518 (65.21%)	589 (55.15%)	
Widowed/Divorced/Separated	1,132 (33.33%)	698 (29.98%)	434 (40.64%)	
Never married	157 (4.62%)	112 (4.81%)	45 (4.21%)	
PIR %				<0.001
Low income	856 (25.21%)	490 (21.05%)	366 (34.27%)	
Med income	1,426 (41.99%)	953 (40.94%)	473 (44.29%)	
High income	1,114 (32.80%)	885 (38.02%)	229 (21.44%)	
Alcohol use %				<0.001
Never	1,005 (29.59%)	611 (26.25%)	394 (36.89%)	
Former	301 (8.86%)	197 (8.46%)	104 (9.74%)	
Mild	1,545 (45.49%)	1,125 (48.32%)	420 (39.33%)	
Moderate	308 (9.07%)	236 (10.14%)	72 (6.74%)	
Heavy	237 (6.98%)	159 (6.83%)	78 (7.30%)	
Smoking %				0.021
No	1,673 (49.26%)	1,178 (50.60%)	495 (46.35%)	
Yes	1723 (50.74%)	1,150 (49.40%)	573 (53.65%)	
Diabetes %				<0.001
No	2,504 (76.72%)	1889 (83.99%)	615 (60.59%)	
Yes	760 (23.28%)	360 (16.01%)	400 (39.41%)	
COPD %				<0.001
No	1,456 (93.09%)	992 (96.31%)	464 (86.89%)	
Yes	108 (6.91%)	38 (3.69%)	70 (13.11%)	
Cardiovascular disease %				<0.001
No	2,666 (78.74%)	2031 (87.39%)	635 (59.79%)	
Yes	720 (21.26%)	293 (12.61%)	427 (40.21%)	
BMI (Kg/m^2^)	29.04 ± 5.96	28.13 ± 5.16	31.01 ± 7.02	<0.001
WC (cm)	102.34 ± 14.51	100.23 ± 13.32	106.93 ± 15.89	<0.001
SBP (mmHg)	132.38 ± 19.88	131.88 ± 18.92	133.48 ± 21.81	0.029
DBP (mmHg)	66.51 ± 13.97	67.46 ± 13.60	64.42 ± 14.55	<0.001
HEI-2015	54.50 ± 12.11	55.40 ± 12.33	52.53 ± 11.35	<0.001
Energy (kcal)	1845.24 ± 782.25	1873.98 ± 773.33	1781.81 ± 798.30	0.002
VAI	0.40 ± 0.70	0.35 ± 0.69	0.52 ± 0.71	<0.001
LAP	3.85 ± 0.70	3.77 ± 0.69	4.01 ± 0.70	<0.001

Continuous variables were summarized using means with SE, and categorical variables were presented as proportions with SE. The p-value indicates the comparison between the frailty and non-frailty groups. COPD, chronic obstructive pulmonary disease; BMI, body mass index; WC, waist circumference; SBP, systolic blood pressure; DBP, diastolic blood pressure; VAI: Visceral Adiposity Index, LAP: Lipid Accumulation Product, PIR: income to poverty ratio.

### 3.2 Multivariate regression analysis

Table 2 compiles the results of the weighted multivariate logistic regression study that assessed the relationship between frailty and VAI and LAP separately. In line with the findings of LAP, it was discovered that the risk of FI prevalence increased with the size of the VAI group. When compared to individuals with lower VAI or LAP index levels, those with higher levels were linked to a higher chance of prevalence FI. In fully adjusted model 3, the odds of frailty increased by 49% for each 1-unit increase in VAI (OR: 1.49, 95% CI: 1.26, 1.77; *p* < 0.0001). The odds of frailty increased by 88% for each 1-unit increase in LAP (OR: 1.88, 95% CI: 1.55, 2.29; *p* < 0.0001). When categorizing VAI, the higher VAI group had a substantially greater prevalence of frailty than the lowest group (OR = 1.92, 95% CI: 1.41, 2.61; *p* = 0.0001 for the highest quartile). The higher LAP group had a substantially greater prevalence of frailty than the lowest group when LAP was categorized by category (OR = 2.64 95% CI: 1.87, 3.74; *p* < 0.0001 for the highest quartile), with a *p* value of ≤0.0001 for all trends.

**Table 2:** Association between VAI/LAP and frailty.

	OR^a^ (95%CI^b^) *p*-value		
	Model 1^c^	Model 1^d^	Model 1^e^
VAI			
Continuous	1.63 (1.40, 1.91)	1.56 (1.34, 1.81)	1.49 (1.26, 1.77)
*P* for trend	<0.0001	<0.0001	<0.0001
Categories			
Quartile 1 (−1.84 < VAI ≤ −0.09)	Reference	Reference	Reference
Quartile 2 (−0.09 < VAI ≤ 0.40)	1.25 (0.91, 1.71)	1.16 (0.85, 1.58)	1.09 (0.78, 1.51)
Quartile 3 (0.40 < VAI ≤ 0.88)	1.83 (1.39, 2.42)	1.62 (1.19, 2.20)	1.42 (1.02, 1.99)
Quartile 4 (0.88 < VAI ≤ 2.45)	2.32 (1.74, 3.09)	2.10 (1.59, 2.79)	1.92 (1.41, 2.61)
*P* for trend	<0.0001	<0.0001	0.0001
LAP			
Continuous	1.83 (1.54, 2.18)	1.92 (1.61, 2.29)	1.88 (1.55, 2.29)
*P* for trend	<0.0001	<0.0001	<0.0001
Categories			
Quartile 1 (0.94 < LAP ≤ 3.39)	Reference	Reference	Reference
Quartile 2 (3.39 < LAP ≤ 3.86)	1.16 (0.83, 1.62)	1.10 (0.78, 1.56)	1.09 (0.761, 1.56)
Quartile 3 (3.86 < LAP ≤ 4.33)	1.48 (1.07, 2.03)	1.44 (1.00, 2.07)	1.29 (0.88, 1.90)
Quartile 4 (4.33 < LAP ≤ 5.84)	2.70 (1.98, 3.68)	2.83 (2.07, 3.86)	2.64 (1.87, 3.74)
*P* for trend	<0.0001	<0.0001	<0.0001

OR^a^, odds ratio; 95% CI^b^, 95% confidence interval; Model1^c^, adjusted for non covariates; Model2^d^, adjusted for age, gender, race, and education; Model3^e^, further adjusted for marry, poverty income ratio, smoking, alcohol use, systolicblood pressure, diastolic blood pressure, healthy eating index-2015 and energy intake.

### 3.3 Nonlinear analysis

**Figure 2:**
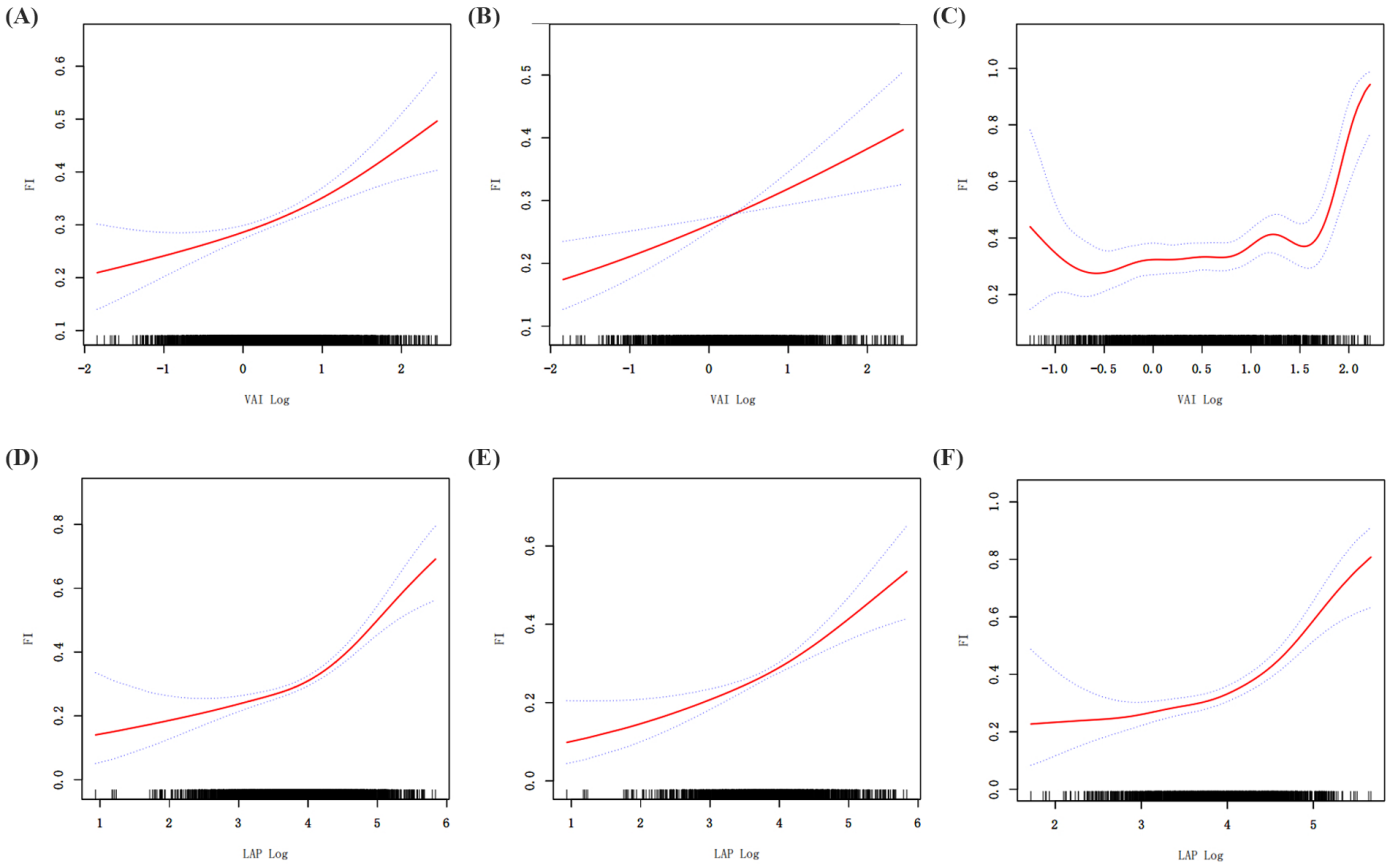
Generalized additive regression. **(A)** GAM for total population VAI; **(B)** GAM for male VAI; **(C)** GAM for female VAI. **(D)** GAM for total population LAP; **(E)** GAM for male LAP; **(F)** GAM for female LAP.

When GAM was used to further evaluate the relationship between VAI, LAP, and frailty, it revealed a significant nonlinear link (Figure 2; Table 3). The existence of a threshold effect was further supported by segmented regression analysis. The inflection points for VAI in the total and female populations are 1.57 and 1.62. The inflection points for LAP in the total and female populations are 4.33 and 4.68. But more investigation showed that genders differed. With a notable threshold effect, the nonlinear effect was noticeable in females. There was no threshold impact, however the prevalence of frailty in males rose with VAI and LAP.

**Table 3:** Segmented regression results.

	OR^a^ (95%CI^b^) *p*-value		
	Total	Males	Females
VAI			
Segmented Model			
Turning point (K)	1.57	1.44	1.62
<K OR 1	1.27 (1.11, 1.46)	1.36 (1.11, 1.66)	1.18 (0.98, 1.43)
	<0.001	0.0029	0.670
>K OR 2	3.83 (1.43, 10.27)	1.03 (0.36, 2.94)	114.86 (10.73, 1229.47)
	<0.001	0.959	<0.001
OR 2–1	3.00 (1.06, 8.49)	0.76 (0.24, 2.36)	97.11 (8.54, 1104.83)
	0.038	0.632	<0.001
Likelihood ratio test	0.037	0.631	<0.001
LAP			
Segmented Model			
Turning point (K)	4.33	4.22	4.68
<K OR 1	1.40 (1.18, 1.66)	1.38 (1.07, 1.79)	1.51 (1.22, 1.86)
	<0.001	0.015	<0.001
>K OR 2	3.23 (2.20, 4.75)	2.34 (1.49, 3.68)	13.85 (4.16, 46.15)
	<0.001	<0.001	<0.001
OR 2–1	2.31 (1.42, 3.75)	1.70 (0.93, 3.10)	9.18 (2.52, 33.46)
	<0.001	0.087	<0.001
Likelihood ratio test	<0.001	0.088	<0.001

OR^a^, odds ratio; 95% CI^b^, 95% confidence interval.

### 3.4 Subgroup analysis

We performed subgroup analyses that considered age, gender, race, education, marital status, BMI, PIR, HEI-2015, smoking, and alcohol use in order to better examine the relationship between VAI and LAP and frailty in various groups. Where age groupings were determined based on the median of the sample. The results of the analysis showed that the risk of FI prevalence in different groups was consistently positively correlated with both VAI and LAP. There were no statistically significant interaction tests in most subgroups, which further strengthens the evidence that VAI and LAP are independent risk factors for FI, respectively (Tables 4, 5).

**Table 4:** Subgroup regression results of VAI.

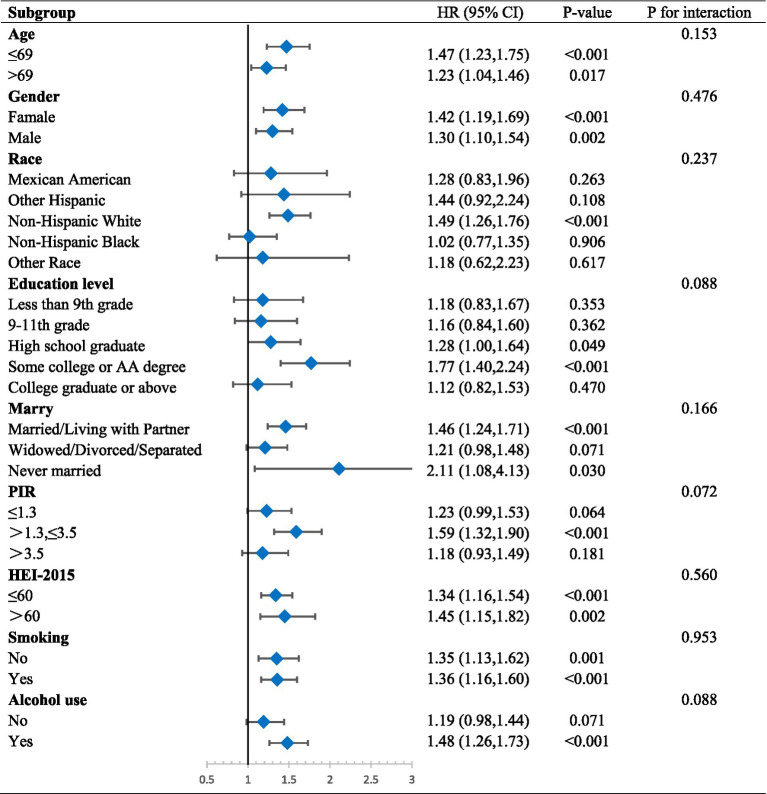

Results of the subgroup analysis were adjusted for all covariates except the effect modifier.

**Table 5:** Subgroup regression results of LAP.

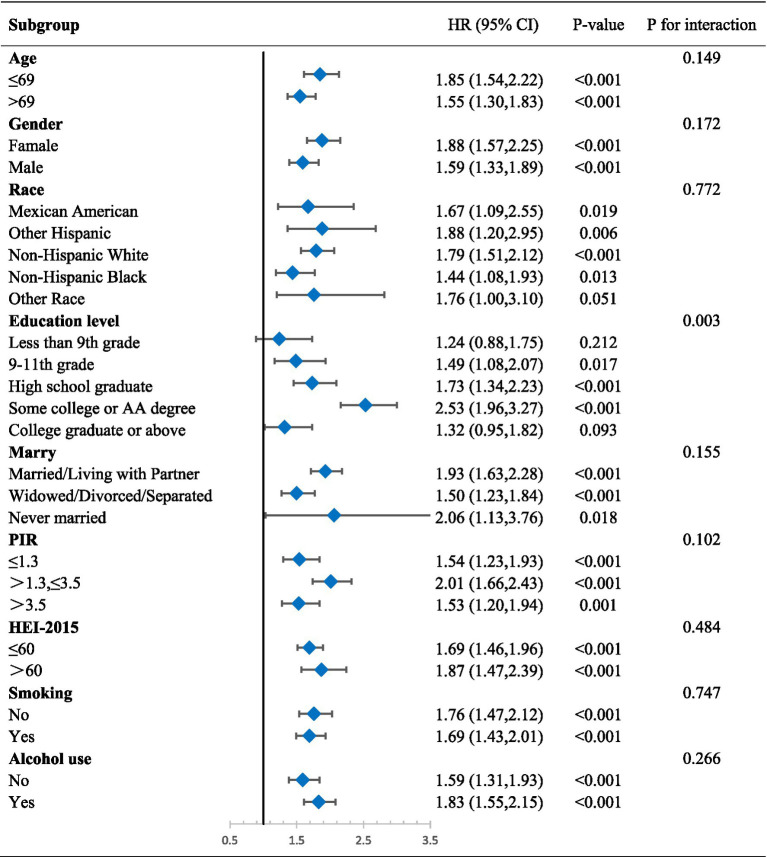

Results of the subgroup analysis were adjusted for all covariates except the effect modifier.

### 3.5 Sensitivity analysis

Several sensitivity assessments were performed, such as estimating missing covariates and correcting for diabetes, chronic obstructive pulmonary disease, cardiovascular illness, and chronic kidney disease, in order to further confirm the data’s robustness. In addition, it was considered that older adults often suffer from multiple chronic diseases. We introduced the age-adjusted Charlson Comorbidity Index to quantify the comorbidity burden of patients, which was calculated by assigning the appropriate weight score to each disease, plus the patient’s age group score (31). We additionally included the Charlson Comorbidity Index for sensitivity analysis. The results of the sensitivity analyses consistently supported significant associations between VAI and LAP and frailty, indicating good stability of the results (Table 6).

**Table 6:** Further adjustment for covariates, disease, and age-adjusted charlson comorbidity index.

	OR^a^ (95% CI^b^) *p*-value		
	Model 4^c^	Model 5^d^	Model 6^e^
VAI			
Continuous	1.47 (1.24, 1.73)	1.30 (1.01, 1.68)	1.39 (1.10, 1.75)
*P* for trend	< 0.001	0.058	0.018
Categories			
Quartile 1	Reference	Reference	Reference
Quartile 2	1.12 (0.83, 1.53)	1.01 (0.58, 1.74)	0.89 (0.51, 1.53)
Quartile 3	1.47 (1.06, 2.02)	1.58 (0.90, 2.78)	1.31 (0.76, 2.27)
Quartile 4	1.88 (1.38, 2.57)	1.64 (1.03, 2.61)	1.83 (1.18, 2.82)
*P* for trend	< 0.001	0.029	0.013
LAP			
Continuous	1.83 (1.51, 2.21)	1.65 (1.24, 2.20)	1.75 (1.33, 2.30)
*P* for trend	< 0.001	0.003	0.002
Categories			
Quartile 1	Reference	Reference	Reference
Quartile 2	1.10 (0.77, 1.57)	1.21 (0.71, 2.06)	1.21 (0.73, 2.01)
Quartile 3	1.30 (0.89, 1.90)	1.42 (0.73, 2.74)	1.22 (0.65, 2.31)
Quartile 4	2.60 (1.85, 3.67)	2.33 (1.39, 3.90)	2.42 (1.50, 3.92)
*P* for trend	< 0.001	0.007	0.008

OR^a^: odds ratio; 95% CI^b^: 95% confidence interval; Model 4^c^: further adjustment for missing covariates; Model 5^d^: further adjustment for diabetes, CVD, COPD, CKD; Model 6^e^: further adjustment for age-adjusted charlson comorbidity index.

### 3.6 ROC analysis

**Figure 3:**
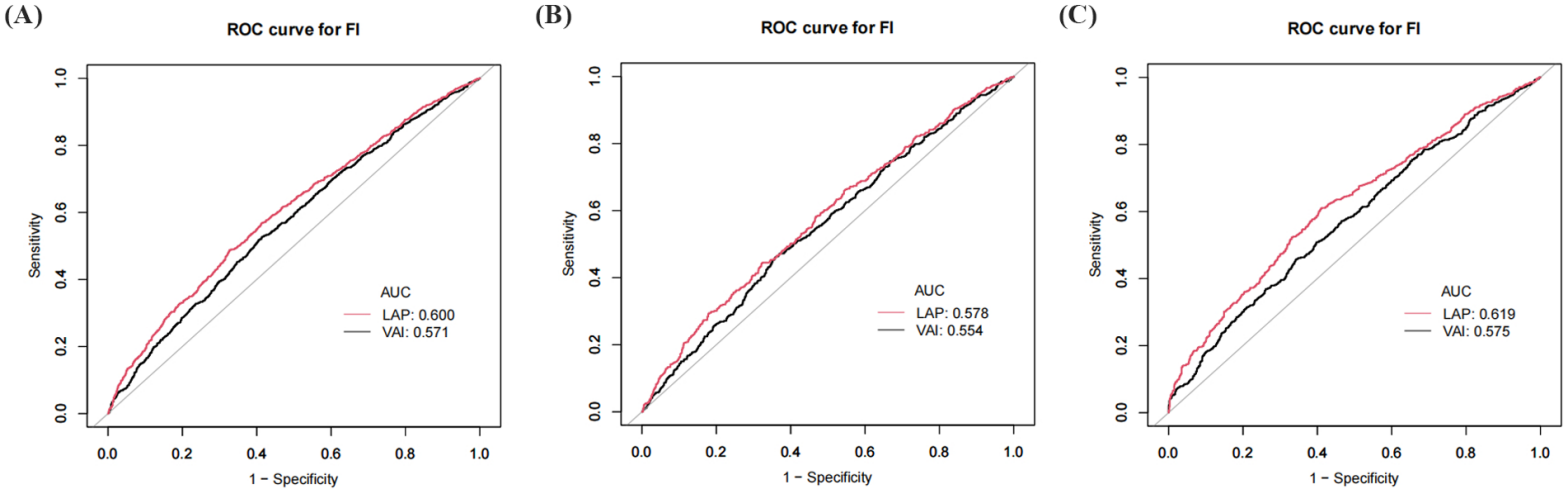
ROC curve of VAI and LAP. **(A)** ROC for total population; **(B)** ROC for males; **(C)** ROC for females. VAI: Visceral Adiposity Index; LAP: Lipid Accumulation Product.

The predictive ability of VAI and LAP for frailty was assessed by ROC analysis. The results showed that the AUC area of LAP was significantly greater than that of VAI in all populations. LAP was a better predictor of frailty than VAI in all populations (Figure 3; Table 7), and there was a significant difference.

**Table 7:** ROC analysis results.

	Variable	AUC (95% CI)	Threshold	Sensitivity	Specificity	Youden Index	*p* value
Total	VAI	0.57 (0.55, 0.59)	1.66	0.52	0.59	0.11	-
	LAP	0.60 (0.58, 0.62)	59.03	0.49	0.67	0.16	<0.001
Males	VAI	0.55 (0.52, 0.58)	1.66	0.46	0.64	0.1	-
	LAP	0.58 (0.55, 0.61)	59.22	0.44	0.68	0.12	0.003
Females	VAI	0.58 (0.55, 0.60)	2.03	0.46	0.66	0.12	-
	LAP	0.62 (0.59, 0.65)	50.65	0.61	0.59	0.2	<0.001

VAI: Visceral Adiposity Index; LAP: Lipid Accumulation Product; WC, waist circumference.

## 4 Discussion

Using NHANES data from 2007–2018, this study assessed the relationships between VAI, LAP, and the risk of prevalent frailty in older persons in the U.S. who are 60 years of age or older. The findings indicated a substantial and independent relationship between VAI and LAP and a higher probability of prevalent frailty. According to fully adjusted models, the risk of prevalent frailty increased by 49% for every unit rise in VAI and by 88% for every unit increase in LAP. The nonlinear link between VAI, LAP, and frailty was made clear by a GAM analysis. When VAI > 1.57 and LAP > 4.33, the probability of frailty increased by 283 and 223%, respectively, for every unit rise in VAI and LAP. The results of the ROC curve analysis showed that LAP outperformed VAI in the prediction of risk of prevalence frailty. Compared to VAI, LAP’s AUC value was substantially greater (*p* < 0.05), further demonstrating the superiority of LAP in distinguishing high-risk populations. In addition, to improve the results’ robustness, this study conducted sensitivity and subgroup analyses. The findings showed considerable stability as the relationships between VAI and LAP and the risk of prevalent frailty remained significant after adjusting for disease covariates. In subgroup analyses, there were constant positive correlated between VAI and LAP and the risk of prevalence FI in different groups, with no statistically significant interaction tests in most subgroups, which further strengthens the evidence that VAI and LAP are independent risk factors for FI, respectively.

The relationship between frailty and obesity, body fat distribution, and metabolic health has been a hot research topic in recent years. For example, Peng et al. found that they were positively associated with the prevalence of NAFLD (32). Li et al. noted that VAI and LAP are key predictors of metabolic syndrome (33). High VAI and LAP significantly increased the risk of metabolic disorders. Yuan et al. found through a systematic review and meta-analysis that abdominal obesity was significantly associated with frailty in older adults and that BMI had a U-shaped relationship with frailty (34), suggesting that either lower or higher BMI may increase the risk of frailty in older adults. We generally understand that for excessively low BMI, frailty may be due to malnutrition, loss of muscle mass, and so on. In contrast, our study reveals for the first time that VAI and LAP are nonlinearly and positively associated with frailty in the elderly, with inflection points of 1.57 and 4.33, respectively. The reason that the present study does not reflect a U-shaped relationship is the high BMI of the included subjects, with a mean value of 29.04, and a severe lack of low-weight subjects. Therefore, the results of the study mainly reflect the health status of the overweight or obese group. In addition, the present study further validated that both VAI and LAP have significant advantages in predicting frailty, especially LAP has better predictive ability than VAI in total population, male or female population. This may be caused by the difference in their calculations, which additionally incorporates BMI and HDL indexes in the calculation of VAI. Changes in the ratio of fat to muscle mass in the elderly population make it possible that BMI may not accurately assess visceral fat (35), thus affecting the accuracy of the VAI. Moreover, HDL usually tends to decline in the elderly population (36). The use of HDL as a denominator in the calculation magnifies its effect on VAI, which in turn affects the accuracy of VAI in predicting frailty. LAP provides a more direct and reliable prediction of frailty in older adults by measuring visceral fat directly from waist circumference and triglycerides. Although the VAI is slightly less accurate than the LAP in predicting frailty in older adults, the VAI provides additional information by combining lipid levels and body fat distribution. The complementary nature of the two in the prediction of frailty risk provides strong evidence for early screening for future frailty in older adults. Frailty is usually a progressive process and may lack obvious clinical symptoms in the early stages, but accumulation of visceral fat and metabolic abnormalities may have already occurred by this time. Therefore, measurement of biomarkers such as VAI and LAP can identify potential risks before frailty becomes apparent, thus providing an opportunity for early intervention. In addition, their monitoring not only identifies potential trends in frailty, but also allows for a comprehensive risk assessment in conjunction with other factors such as physical activity levels and dietary status. This multi-dimensional risk assessment helps to tailor a more precise health intervention program for the individual, reducing the incidence of frailty and slowing its progression. It is important to emphasize that further consideration of factors such as mortality and hospitalization rates would help to more fully assess the value of the clinical application of VAI and LAP in frailty screening.

Obesity, particularly the accumulation of abdominal fat, increases the risk of frailty through multiple pathophysiologic processes. The metabolically active tissue known as abdominal fat can release a number of pro-inflammatory substances, including interleukin-6 and tumor necrosis factor-alpha, which accelerate the loss of muscular mass and strength and cause systemic, persistent, low-grade inflammation, both of which lead to frailty (14). This inflammatory state is thought to be one of the main drivers of weakness, and the onset of weakness may result from the interaction of metabolic disorders triggered by fat accumulation and chronic inflammation. Individuals with high VAI and LAP are usually accompanied by higher abdominal fat stores and are susceptible to the negative effects of inflammatory responses. This chronic low-grade inflammation not only affects muscle mass but may also accelerate multisystem decline through oxidative stress (37). At the same time, there is a substantial correlation between the development of insulin resistance and the accumulation of abdominal fat, which directly contributes to metabolic disorders such as diabetes and hyperlipidemia, and these metabolic disorders further accelerate the progression of frailty (38). In the present study, LAP was found to be a better predictor of frailty than VAI, which may be related to the accurate reflection of abdominal fat by LAP. In addition, elevated VAI and LAP are also closely associated with sarcopenic obesity, a state that is usually accompanied by the coexistence of excess adipose tissue and insufficient muscle mass, leading to decreased muscle function, reduced strength, and metabolic efficiency, which accelerates the onset of frailty (39). In addition, excessive obesity is usually accompanied by higher levels of depression (40), anxiety, and other psychological problems, which may act indirectly by reducing physical activity levels, altering dietary habits, and exacerbating chronic inflammation that further accelerates the onset of frailty (41). Abdominal obesity is associated with several hormonal imbalances, including leptin (42), insulin-like growth factor (IGF-1) (43), and abnormalities in sex hormone binding globulin (SHBG) (44). These hormones are closely related not only to energy metabolism and fat distribution but also have important effects on muscle mass and bone strength. Obese individuals often exhibit higher leptin resistance and lower levels of IGF-1 (45), which may further exacerbate the state of weakness. O’Caoimh et al. (46) found that the prevalence of frailty was higher in women than in men through a systematic evaluation and meta-analysis of frailty prevalence from 62 countries and regions. Our study is in line with it, in the subgroup analysis of VAI and LAP, the risk of frailty was higher in the female population. This may be due to the fact that women experience significant hormonal changes after menopause, especially a decrease in estrogen levels, leading to an increase in visceral fat, a decrease in skeletal muscle mass, and consequently a higher risk of frailty. Moreover, with age, especially in postmenopausal women, the pattern of fat distribution changes and more fat begins to shift to the abdominal and visceral regions (47). In addition, the metabolic characteristics of women, such as insulin resistance, fatty acid metabolism, and loss of muscle mass, may be more sensitive, leading to a more pronounced manifestation of frailty in the face of high VAI or LAP. Of note, it is commonly believed that the risk of frailty increases with age as physiologic functions gradually decline. However, our study found a higher risk of frailty in the 60–69 year old group in subgroup analyses of VAI and LAP, and the interaction test was not significant. With age, the metabolic response may be more sensitive in the 60–69 year old group, leading to a more pronounced early manifestation of frailty, despite the beginning of a gradual decline in metabolic and immune function. Individuals maintain high levels of social and life functioning despite the onset of gradual changes in physical condition, which also leads to early manifestations of frailty being more easily detected (48). In addition, cognitive decline and early mental health problems (e.g., depression, anxiety) may manifest more prominently at this stage, exacerbating the onset of frailty. Comparatively, the 70–80 year old group, although older, may have adapted to some of the changes in the aging process, have more stable physical and metabolic systems, or have taken some health management measures to slow the progression of frailty as much as possible. And in this group, frailty may be more often associated with severe disease, long-term chronic health problems (e.g., cardiovascular disease, diabetes, etc.). The interaction was not significant indicating that the relationship between indicators such as VAI and LAP and frailty did not reach statistical significance in terms of differences between age groups. In other words, the effects of VAI and LAP on frailty showed similar trends across age groups and were not significantly moderated by age group. This implies that the predictive ability of VAI and LAP on frailty was consistent across age groups, although the risk of frailty was higher in the 60–69 age group.

The large and nationally representative sample size based on NHANES data is one of the study’s main strengths, as it increases the validity of the findings. The study adjusted for multiple potential confounders, including age, sex, race, and lifestyle factors, enhancing the reliability of the findings. Subgroup analyses and sensitivity analyses showed that the correlations of VAI and LAP with frailty were significant across populations, further supporting the robustness of the results. The study also had limitations. As the data were from a cross-sectional survey, a causal association between VAI and LAP and frailty could not be established, and further longitudinal studies are needed to validate this. Despite adjusting for multiple variables, there may be potential confounders (e.g., genetic, etc.) that were not captured. The FI index was chosen to assess frailty in this study because it can comprehensively assess the multidimensional characteristics of frailty. However, the Fried phenotype method is widely used due to its greater simplicity and ease of use in the process of frailty assessment. Therefore, it is recommended to try to combine these two methods in future studies, taking into account their respective advantages, in order to further optimize the accuracy and practicality of frailty assessment. In addition, further exploration of important factors affecting frailty such as number of medications, comorbidity burden, and walking speed would make the study more comprehensive and accurate. The study population was an older U.S. population, and more research is needed to confirm whether the findings are applicable to different areas or ethnic groups.

## 5 Conclusion

This study showed that among older persons in the U.S., frailty, LAP, and VAI were significantly positively correlated. The predictive ability of LAP for frailty was superior to that of VAI, revealing the potential of VAI and LAP as emerging indicators for frailty risk prediction, and the finding of a threshold effect, in particular, is of great clinical significance. Further validation in larger prospective studies is still needed in the future.

## Data Availability

Publicly available datasets were analyzed in this study. This data can be found here: https://wwwn.cdc.gov/nchs/nhanes/.
